# A Case of Acute Myeloid Leukemia, Myelodysplasia-Related (AML-MR) with Immunophenotypic Features Suggestive of B/Myeloid Mixed-Phenotype Acute Leukemia (MPAL), Harboring Multiple *KMT2A* Gene Amplifications

**DOI:** 10.3390/ijms27167170

**Published:** 2026-08-11

**Authors:** Jakub Łączak, Marta Szarawarska, Dominika Dudycz, Karolina Bieńko, Jarosław Grzyb, Tomasz Skoczylas, Beata Blajer-Olszewska, Agnieszka Kopacz, Mirosław Markiewicz

**Affiliations:** 1 Department of Hematology, F. Chopin University Clinical Hospital, 35-055 Rzeszów, Poland; 2 Department of Hematology, Faculty of Medicine, Collegium Medicum, University of Rzeszów, 35-310 Rzeszów, Poland

**Keywords:** KMT2A, gene amplification, mixed phenotype acute leukemia

## Abstract

The amplification of *KMT2A*, a gene involved in hematopoietic stem cell function, is extremely rare in acute leukemias, especially in mixed-phenotype acute leukemia. We present the case of a 62-year-old patient diagnosed with acute myeloid leukemia, myelodysplasia-related (AML-MR) with immunophenotypic features suggestive of B/myeloid mixed-phenotype acute leukemia (MPAL), with the presence of multiple *KMT2A* gene amplifications and a very aggressive and complicated clinical course ending in failure despite intensive treatment. *KMT2A* amplification in MPAL could be a risk factor suggesting adverse outcomes.

## 1. Introduction

The *KMT2A* gene, formerly *MLL* (mixed-lineage leukemia), located on chromosome 11q23, encodes a histone methyltransferase involved in the activation of genes regulating hematopoietic stem cell self-renewal and differentiation [1]. While *KMT2A* rearrangements are frequently encountered in acute myeloid leukemia (AML) and B-lineage acute lymphoblastic leukemia (B-ALL), *KMT2A* amplification is extremely rare, occurring in <1% of AML cases [2] and even less frequently in B-ALL (only three documented cases in adults have been described to date, all of which resulted in death within 4–14 days of diagnosis) [3].

Mixed-phenotype acute leukemia (MPAL) occurs in approximately 2–5% of acute leukemia cases and has a rapid and unfavorable natural history [4]. Diagnosis of MPAL requires the demonstration of antigen expression from more than one cell lineage, which may be accompanied by a variety of genetic alterations. To our knowledge, only three cases of MPAL with *KMT2A* amplification have been reported to date [5]. We present the case of a 62-year-old patient diagnosed with AML-MR, with immunophenotypic features suggestive of B-cell/myeloid MPAL and multiple *KMT2A* gene amplifications. The clinical course was highly aggressive, with central nervous system involvement, severe coagulation disorders, infectious complications, features of generalized malignancy, secondary hemophagocytic syndrome, and, consequently, failure despite intensive treatment on the 25th day of hospitalization.

## 2. Case Report

A 62-year-old man presented to the emergency room of a district hospital with complaints of weakness and recurrent fevers lasting approximately one week. A complete blood count (CBC) revealed life-threatening anemia and profound thrombocytopenia (30 G/L). Upper gastrointestinal bleeding was initially suspected, but it was ruled out by gastroscopy. However, a massive, difficult-to-control nosebleed occurred, which was caused by coagulation disorders, including significantly prolonged activated partial thromboplastin time (APTT) and prothrombin time (PT). Substitution therapy was performed using red-blood-cell concentrates (RBCs), platelet concentrates (PCs), and fresh frozen plasma (FFP), and the patient was transferred to the Hematology Clinic with suspicion of acute bone marrow malignancy. The patient’s general condition was severe, with an Eastern Cooperative Oncology Group (ECOG) score of 2, significant weakness and spontaneous skin bruising, the presence of large bruises at various stages of development all over the body, and hepatosplenomegaly (with the liver and spleen 2 cm and 4 cm below the costal margin, respectively). A peripheral blood count revealed pancytopenia: hemoglobin (Hgb), 11.9 g/dL (after an RBC transfusion 2 days earlier); platelets (PLT), 25 G/L; and absolute neutrophil count (ANC), 0.4 G/L, with 5.88 G/L of white blood cells (WBCs). A microscopic peripheral blood smear yielded 47% blasts. Laboratory tests showed elevated lactate dehydrogenase (LDH) levels (1322 U/L); hyperferritinemia (10,347 ng/mL); hypertriglyceridemia (287 mg/dL); high inflammatory parameters, namely, a C-reactive protein (CRP) level of 25.7 mg/dL (upper limit of normal [ULN], 0.5 mg/dL), a procalcitonin (PCT) level of 0.56 ng/mL (ULN, 0.5 ng/mL), and high D-dimers (above 34,000 ng/mL); and signs of acute kidney injury (creatinine, 1.59 mg/dL; urea, 58 mg/dL).

Ultrasound confirmed hepatosplenomegaly (liver craniocaudal diameter [CC], 174 mm; spleen, 140 mm × 49 mm) and minor peripheral lymphadenopathy affecting numerous nodes. A contrast-enhanced whole-body computed tomography (CT) scan revealed intra-abdominal mesenteric fat densities with fat necrosis, consistent with panniculitis.

Due to the clinical presentation (fever, splenomegaly) and laboratory abnormalities (cytopenias, hypertriglyceridemia/hypofibrinogenemia, hyperferritinemia), five of the eight criteria for hemophagocytic syndrome (HLH-2004) were met. Natural killer (NK) cell cytotoxicity and soluble interleukin-2 (IL-2) receptor concentration were not assessed, and the third missing criterion was the absence of hemophagocytosis, which is not strictly required for the diagnosis of HLH. Secondary HLH was diagnosed, and dexamethasone at a dose of 2 × 10 mg/day was therefore included in the treatment. Due to the presence of agranulocytosis and an undetermined infection, empirical broad-spectrum antibiotic therapy (cefepime, teicoplanin) was administered concurrently, resulting in resolution of the fever and a reduction in inflammatory parameters.

**Figure 1:**
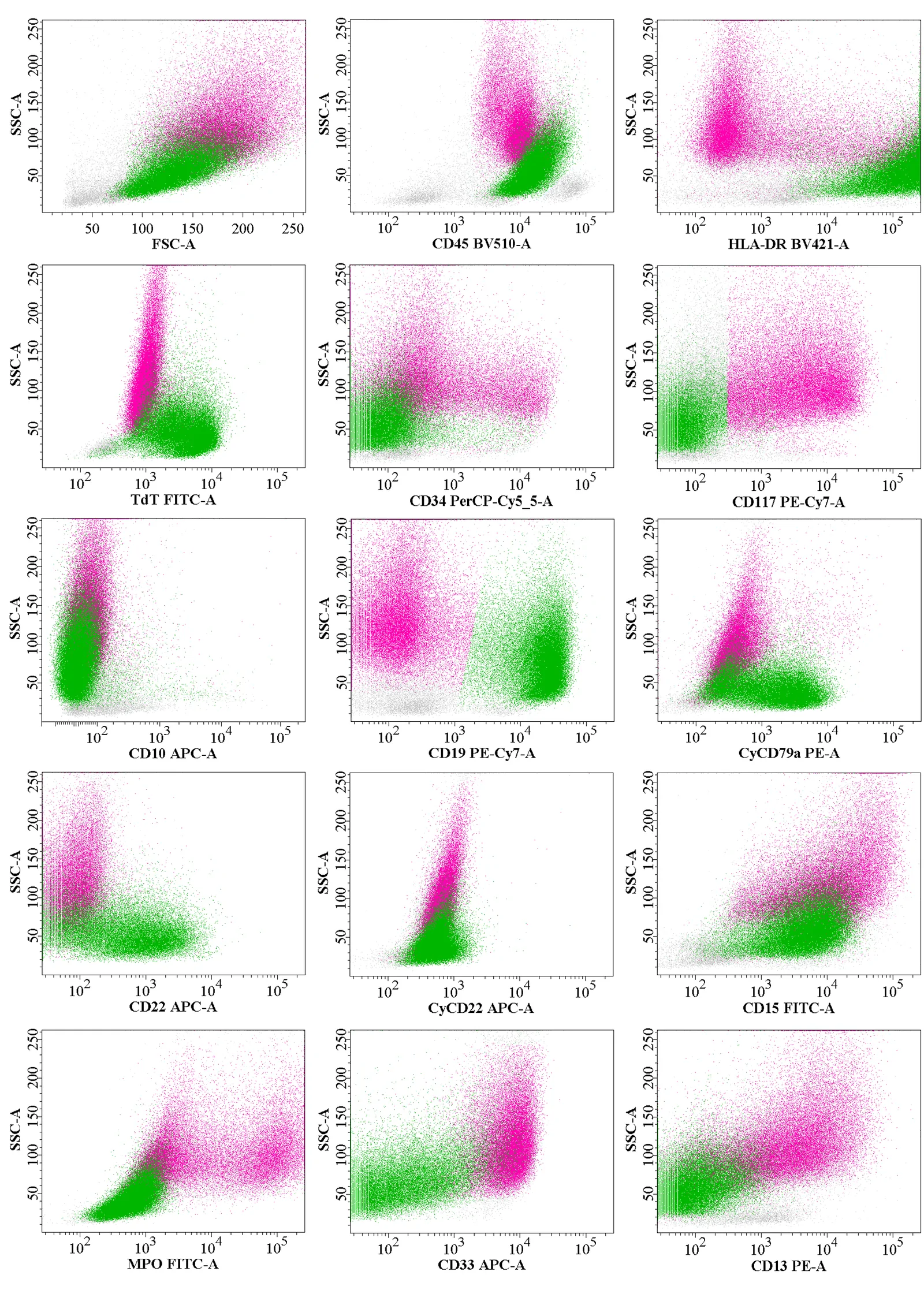
Cytometric analysis of bone marrow. Two antigenically distinct populations of leukemic cells were identified, sharing the common feature of positive expression of the CD15 marker. The first, representing approximately 47.6% of bone marrow nucleated cells (green), showed features of B-lymphoid differentiation (strong expression of CD19, presence of cyCD79a, TdT, and partial expression of sCD22) while lacking myeloid markers (cMPO, CD13, CD33). The markers CD10 and CD34 were also absent among the cells in this population. The second leukemic population was less numerous, comprising approximately 28.1% of bone marrow nucleated cells (pink), and its antigenic characteristics indicated myeloid lineage. The cells of this population showed the presence of the markers CD117, CD13, CD33 (strong expression), and cMPO and the absence of expression of CD34, HLA-DR, and B-lymphoid antigens. The immunophenotype of the second leukemic population was closest to the antigenic profile of cells at the promyelocyte stage. According to the WHO immunophenotypic diagnostic criteria for MPAL, the overall bone marrow findings indicated a diagnosis of acute leukemia with a mixed B/myeloid phenotype.

Myelogram evaluation showed an infiltration of large and medium-sized dysplastic immature cells with single or double nucleoli, with some vacuoles, and numerous shadows of disintegrated cells. Flow cytometry examination of the bone marrow revealed the presence of two immunophenotypically distinct, although morphologically similar, pathological cell populations, suggesting a diagnosis of acute leukemia with a mixed phenotype (MPAL B/myeloid, according to the 2022 WHO classification)—phenotypic details are presented in Figure 1.

Molecular PCR testing excluded the presence of the *BCR::ABL1* and *PML-RARA* fusion genes and pathogenic variants of the nucleophosmin 1 (*NPM1*) and FMS-like tyrosine kinase 3 (*FLT3*) genes (internal tandem duplication [*ITD*] and tyrosine kinase domain [*TKD*] types). Cytogenetic analysis revealed a monosomal karyotype with the presence of 45–46 chromosomes and aberrations involving chromosomes 1, 5, 11, 15, 16, 17, 20, and 21. These aberrations resulted in the formation of a der(11) derivative chromosome and amplification of the genetic material of the long arm of chromosome 11, including the *KMT2A* (*MLL*) locus (11q23), in 46 of 100 (46%) of the interphase cells analyzed, containing up to 18 copies of *KMT2A*, and in three metaphases. Amplification occurred in homogeneously staining regions (HSRs). Furthermore, trisomy of chromosome 8 and monosomy of chromosome 17 were detected in each of the analyzed metaphases.

**Figure 2:**
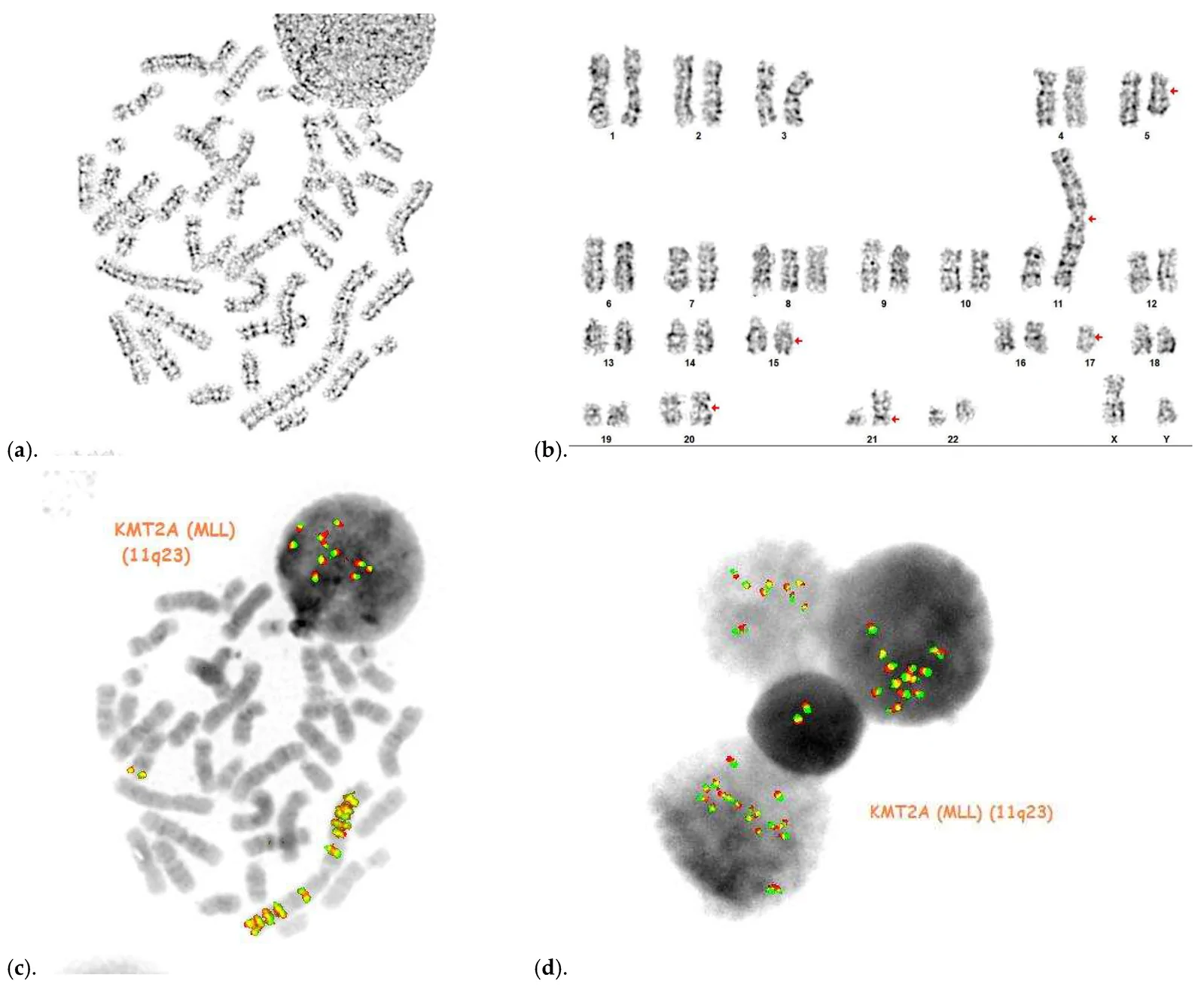
Cytogenetic evaluation (**a**). Metaphase presenting numerous and structural chromosomal abnormalities (**b**). Conventional karyotype showing a complex karyotype, including *homogenously staining region* (*hsr*), i.e., the segment of a chromosome that stains uniformly after G-banding (**c**). Fluorescence in situ hybridization with the XL *MLL* Dual Color Breakapart probe (MetaSystems, Altlussheim, Germany) specific for the *KMT2A* gene (11q23) showing multiple copies of the *KMT2A* gene located in the hsr(11)(11qter → 11q23::hsr → 11p10 → hsr::11q23 → 11qter) chromosome (**d**). Interphase nuclei showing *KMT2A* amplification with >17 signals per cell.

Fluorescent in situ hybridization (FISH) revealed the presence of one chromosome 17 centromere signal (D17Z1) and the TP53 locus (17p13) in one metaphase and in 50 of 100 (50%) interphase cells analyzed. In 19 of 100 cells analyzed, one TP53 signal was detected along with two chromosome 17 centromere signals. This means that FISH detected two cellular subclones: one that was more abundant with monosomy of chromosome 17 and the other with a monoallelic deletion of the TP53 locus. A monosomal karyotype and monosomy 17/TP53 loss are defining myelodysplasia-related cytogenetic abnormalities. The aberrations described above were confirmed with color probes. FISH revealed the presence of three *RARA* and *BCR* gene signals in 17% and 18% of the 100 interphase cells analyzed, respectively. However, no *RARA* locus rearrangement or *BCR* (22q11) or *ABL1* (9q34) gene fusions were detected in any of them. Conventional cytogenetic and FISH evaluations are shown in Figure 2, and detailed karyotype results are presented in Table 1.

Histopathological examination of a representative biopsy specimen revealed bone marrow with 100% cellularity resulting from a diffuse blast infiltrate with a round nucleus, prominent eosinophilic nucleoli, and eosinophilic cytoplasm. The histopathological findings were consistent with a diagnosis of acute leukemia, which more closely resembled acute myeloid leukemia. The antigenic characteristics of the leukemic cell infiltrate in the biopsy specimen are presented in Table 2.

Cerebrospinal fluid examination revealed central nervous system (CNS) involvement corresponding to a 6% myeloid clone of leukemic cells, with a pleocytosis of 3 cells/mcL. The patient received intrathecal triplet chemotherapy (methotrexate (15 mg), cytarabine (40 mg), and dexamethasone (4 mg)).

During hospitalization, a gradual increase in coagulation abnormalities was observed: a decreased fibrinogen concentration (to a minimum of 85 mg/dL), and prolongation of aPTT (maximum: 82.5 s) and PT (maximum: 13.6 s). D-dimer levels, after a transient decrease, rose again to >34,000 ng/mL. Chronic bleeding from the central venous catheter site was observed, and treatment included replacement transfusions of FFP, cryoprecipitate, PCs, and antifibrinolytic drugs. Due to the diagnosis of AML with a poor prognosis, intensive induction therapy was administered according to the CLAG-M protocol (Cladribine, Ara-C, G-CSF, Mitoxantrone). Starting on day 2 of chemotherapy (day 10 of hospitalization), fevers returned, and inflammatory parameters re-elevated. Antibiotic therapy (meropenem, vancomycin) and antifungal therapy (caspofungin) were escalated, initially with good results, but less than two weeks later, septic shock caused by vancomycin- and aminoglycoside-resistant *Enterococcus faecium* (HLAR, VRE) occurred. A further modification of the escalated anti-infective therapy was implemented (linezoid, cortimoxazole, amphotericin B), along with intravenous immunoglobulin replacement and pressor amine infusion. Progressive liver failure was observed, accompanied by renal failure, and on the 17th day of treatment, cardiac arrest and death from asystole occurred. Posthumously, the patient’s blood culture showed the presence of the fungus *Saprochaete capitata*, which is resistant to caspofungin and sensitive to amphotericin B.

## 3. Discussion

Mixed-phenotype leukemias are a heterogeneous group of diseases encompassing both malignancies characterized by the simultaneous expression of antigens from two lineages and those in which two (or three) populations of blast cells from different lineages coexist [6,7].

According to the fifth edition of the WHO Classification of Hematolymphatic Tumors (WHO-HAEM5), MPAL and acute leukemia of unknown origin (ALAL) are grouped together in the same category due to overlapping clinical and immunophenotypic features and shared molecular pathogenetic mechanisms. This classification takes into account molecular abnormalities that separate MPAL/ALAL subtypes with defined genetic alterations from those diagnosed based on immunophenotype [8].

Although the immunophenotype suggested B/myeloid mixed-phenotype acute leukemia (MPAL), the final diagnosis was acute myeloid leukemia, myelodysplasia-related (AML-MR), according to the WHO classification. The presence of defining myelodysplasia-related cytogenetic abnormalities, including a complex monosomal karyotype and monosomy 17/TP53 loss, takes diagnostic precedence over immunophenotypic findings.

In the described case, *KMT2A* gene amplification was detected, an extremely rare phenomenon that may indicate an exceptionally poor prognosis, although this observation is based on limited case reports. This gene encodes a transcriptional coactivator that plays a crucial role in regulating gene expression in early human ontogenetic development and hematopoiesis. Functionally, the protein, a histone methyltransferase (a SET domain enzyme), causes methylation of histone H3 lysine 4 (H3L4), resulting in epigenetic activation of transcription, including with respect to many *Hox* genes. Multiple chromosomal translocations involving this gene cause some acute leukemias, and alternate splicing leads to the generation of multiple transcript variants. *KMT2A* is therefore an epigenetic regulator of homebox genes, which directly regulate cell development and differentiation. In the context of leukemia pathogenesis, oncoproteins produced by *KMT2A* mutations block differentiation of progenitor cells and lead to their uncontrolled proliferation. These cells often retain multipotency, which explains their mixed, multilineage phenotype. However, it should be noted that the effects of KMT2A fusion proteins must be clearly distinguished from the accumulation of wild-type KMT2A protein. *KMT2A* mutations cause approximately 75% of acute leukemias in infants (<1 year of age) and 5–10% of AML and ALL in children (>1 year of age) and adults [9]. The age distribution of MPAL with *KMT2A* mutations is similar: among the 61 total identified cases, 35, 16, and 10 were infants, children, and adults, respectively [10].

Recently, Pantrangi et al. [5] described three patients with MPAL and *KMT2A* gene amplification and demonstrated that this rare genetic alteration is associated with an adverse clinical outcome. Their findings indicated that *KMT2A*-amplified MPAL is characterized by a complex karyotype and concurrent TP53 aberrations. Consistent with these observations, the patient described in our report also exhibited a highly complex karyotype, exhibiting a monosomal karyotype. Notably, the *KMT2A* amplification in our case was present on a rearranged chromosome 11, mirroring case 3 reported by Pantrangi et al., whereas the remaining cases showed amplification involving a ring chromosome 7 or multiple marker chromosomes. Importantly, our patient also demonstrated abnormalities involving chromosome 17 and the TP53 locus, with two cytogenetic subclones showing monosomy 17 or monoallelic TP53 deletion.

Takeda et al. reported another interesting case of uncontrollable leukemia with consecutive lineage switches (initially B-ALL, later B/T bi-phenotypic, and finally complete lineage conversion to AML), with an unstable complex karyotype and an increased number of *KMT2A* genes [11].

Diagnosing MPAL can be difficult, especially when immunophenotypic findings are inconsistent with the histopathological picture, as was the case with the patient discussed here, for whom the diagnosis was based on cytological and cytometric evaluation of the bone marrow and molecular testing, while the histopathological evaluation of the biopsy specimen was still being processed at the time. The histopathological results obtained after revealing the immunophenotypic pattern of MPAL were more consistent with acute myeloid leukemia. This may be explained by the lower sensitivity of histopathological assessment, which is most appropriate when a dry biopsy is obtained by aspiration. The difference in the percentages of individual cell populations could be due to differences in preparation. The architectural characteristics of the infiltrate could also be important. However, flow cytometry is the method of choice for diagnosis due to its short turnaround time, ability to quantify the percentage of blasts, and simultaneous detection of multiple antigens using various fluorochrome-labeled antibodies [12]. In the case discussed, CNS involvement was limited to the myeloid lineage. Flow cytometry revealed that 6% of cells had a CD34−/CD117+/CD64+dim/CD38+/CD15+/CD33+high immunophenotype, consistent with the bone marrow immunophenotype.

The choice of an intensive yet safe induction treatment using a hybrid protocol based on cladribine, cytarabine, granulocyte colony-stimulating factor (G-CSF), and mitoxantrone (CLAG-M) was based on the favorable experience of the Polish Adult Leukemia Group (PALG), which showed that all MPAL patients treated with the CLAG-M regimen as first-line induction therapy achieved remission, with no deaths or serious complications reported. It should be noted, however, that although three-quarters of these patients had cytogenetic aberrations and genetic mutations, none of them had abnormalities in the *KMT2A* gene [13].

The contribution of *KMT2A* amplification to leukemogenesis remains uncertain, and its role as a driver or passenger alteration has yet to be established. In conclusion, *KMT2A* amplification in MPAL could be a risk factor indicating adverse outcomes.

## Figures and Tables

**Table 1:** Karyotype.

Number of Chromosomes	Aberrations that Occurred	Number of Metaphases Examined
45~46, XY	der(5)(5pter → 5q22::5q35 → 5qter), +8, hsr(11)(11qter → 11q23::hsr → 11p10 → hsr::11q23 → 11qter), der(15)(15pter → 15q21::16q13 → 16q24::15q21 → 15qter), der(16)(16pter → 16q13::20q12 → 20qter), −17, der(20)(20pter → 20q12::17q21 → qter), der(21)(1pter → 1p34::21pter → 21qter)	30

**Table 2:** Antigenic characterization of leukemia cell bone marrow infiltration in an immunohistochemical examination of a trephine biopsy specimen.

Immunological Phenotype	Comments
CD10−, CD117+ (70% of cells), CD13−, CD138−, CD20−, CD3−, CD33+ (95% of cells), CD34+ (5% of cells), CD61−, CD71+, CD99+ (most cells), MPO+ (70% of cells), TDT+ (some cells)	CD71 expression on numerous blasts, with negative staining for E-cadherin

## Data Availability

The original contributions presented in this study are included in the article. Further inquiries can be directed to the corresponding author.
